# Innovative Sensor Approach to Follow *Campylobacter jejuni* Development

**DOI:** 10.3390/bios9010008

**Published:** 2019-01-07

**Authors:** Estefanía Núñez-Carmona, Marco Abbatangelo, Veronica Sberveglieri

**Affiliations:** 1 Department of Information Engineering, University of Brescia, Brescia, via Branze, 38, 25123 Brescia, BS, Italy; e.nunezcarmona@unibs.it; 2 CNR-IBBR, Institute of Bioscience and Bioresources, via Madonna del Piano, 10, 50019 Sesto Fiorentino, FI, Italy; veronica.sberveglieri@ibbr.cnr.it; 3 NANO SENSOR SYSTEMS, NASYS Spin-Off University of Brescia, Brescia, via Camillo Brozzoni, 9, 25125 Brescia, BS, Italy

**Keywords:** *Campylobacter jejuni*, VOCs, GC-MS SPME, nanowire sensors, PCA

## Abstract

*Campylobacter spp* infection affects more than 200,000 people every year in Europe and in the last four years a trend shows an increase in campylobacteriosis. The main vehicle for transmission of the bacterium is contaminated food like meat, milk, fruit and vegetables. In this study, the aim was to find characteristic volatile organic compounds (VOCs) of *C. jejuni* in order to detect its presence with an array of metal oxide (MOX) gas sensors. Using a starting concentration of 10^3^ CFU/mL, VOCs were analyzed using Gas-Chromatography Mass-Spectrometry (GC-MS) with a Solid-Phase Micro Extraction (SPME) technique at the initial time (T0) and after 20 h (T20). It has been found that a Campylobacter sample at T20 is characterized by a higher number of alcohol compounds that the one at T0 and this is due to sugar fermentation. Sensor results showed the ability of the system to follow bacteria curve growth from T0 to T20 using Principal Component Analysis (PCA). In particular, this results in a decrease of ΔR/R_0_ value over time. For this reason, MOX sensors are a promising technology for the development of a rapid and sensitive system for *C. jejuni*.

## 1. Introduction

Nowadays we live in the safest environment regarding the food industry. Organizations as EFSA (European Food Safety Agency), WHO (World Health Organization), and FAO (Food and Agriculture Organization) determine, organize and rule all the regulations that control every single aspect of food safety, security and trading. Even if the risk perception regarding the food industry is not one of the first concerns of the population in non-developing countries [1,2], food poisoning is still the first cause of hospitalization in the world. The CDC (Center of Disease Control) estimates that each year 48 million people get sick from a foodborne illness, 128,000 are hospitalized and 3000 die just in the USA [3]. 

The FoodNet (Foodborne Diseases Active Surveillance Network) division of the CDC affirms in its 2017 report that the incidence of infections per 100,000 people was highest for *Campylobacter spp* and *Salmonella spp* from 2016 to 2017, which is similar to previous years. The situation in Europe for *Campylobacter spp* infections is illustrated by the Campylobacteriosis-Annual epidemiological report published the 30 January of 2017 by the ECDC (European Center of Disease Control) from data collected in 2014 [4]. The report affirms that 240,379 confirmed cases were reported in 2014 with a rate of campylobacteriosis of 59.8 cases per 100,000 population in the EU/EEA, representing a 13% increase compared with the previous year. Human campylobacteriosis was more common in children below five years of age and in general was slightly higher for males than females across all age groups. Campylobacteriosis shows a clear seasonality, with a sharp peak of cases in July, trend that it is confirmed by the CDC as well. At the beginning of the summer of 2018 in Pescara (Italy), 180 cases of intoxicated children were reported and identified as campylobacteriosis infection. 

The most representative etiologic agent for campylobacteriosis is *Campylobacter jejuni*. It is a slender, spirally curved rod that possesses a single polar flagellum at one or both ends of the cell. It is oxidase and catalase positive, is microaerophilic, requiring small amounts of oxygen (3–6%) for growth; its optimum growth temperatures on solid media are 37 °C, and it grows well at pH 5.5–8.0. *C. jejuni* is associated with warm-blooded animals, in fact a large percentage of all major livestock animals have been shown to contain these organisms in their feces [5,6]. Some strains of *C. jejuni* produce a thermolabile enterotoxin (CJT), that has been reported to have some similar properties with the enterotoxins of *Vibrio cholerae* (CT) and *Escherichia coli* (LT) [7,8]. 

Many livestock warm-blooded animals can carry in their intestines, liver and giblets cells of *C. jejuni* that can be transferred to other edible parts of an animal when it’s slaughtered. In the USA in 2014, National Antimicrobial Resistance Monitoring System (NARMS) testing found *C. jejuni* on 33% of raw chicken bought from retailers [9]. *Campylobacter spp* infection can also be transmitted through unpasteurized milk ingestion when a cow has *C. jejuni* cells in its udder or when milk is contaminated with manure [10]. Moreover, most problematic foods regarding *Campylobacter spp* infection are those consumed raw, such as fruits and vegetables that can be contaminated through contact with soil containing feces from cows, birds or other animals [11]. Animal feces can also contaminate water sources, such as lakes and streams. 

Today there are many techniques that can be used in the identification of this type of contamination, many of which have limits related to the collection time of responses, high complexity or the possibility of being reused several times.

In the last few years, different kinds of sensors have been developed for *C. jejuni* detection. They are essentially DNA-biosensors, of which specificity is due to oligonucleotide probes covalently immobilized on the sensing surface. Several optical [12], acoustic [13] and electrochemical [14] techniques have been proposed for traducing the hybridization with the specific target nucleic acid to the pathogen detection [15]. As an example, quartz crystal microbalance (QCM) immunosensors, using monoclonal and polyclonal antibody systems coupled with the use of gold nanoparticles (AuNPs) to increase the sensitivity, were used [16]. In this way, a limit of detection (LOD) of 10^4^ CFU/mL was reached; however, this kind of sensor is limited to a single use and consequently does not have a low usage cost. The same LOD was reached using a colorimetric aptasensor, that can be used for on-line applications and gives its response in 30 min [17].

As an example, to overcome time consumption and single-use limitations, approaches based on nanowire gas sensor technology could be employed. Nanowire gas sensors base their action principle on the analysis and recognition of the volatile fingerprint of a determinate sample. This kind of approach has already been applied successfully in many different fields as human microbiota monitoring [18], and environmental monitoring [19,20]. In particular, regarding food microbial contamination, nanowire tech was able to recognize the presence of a determinate microorganism throughout the set of volatile organic compounds (VOCs) emitted when growing in a determinate matrix [21,22,23]. In comparison with the aforementioned sensor technologies, nanowire gas sensors exhibit advantages of nanostructured materials such as long-term stability for sustained operations, high rate surface/area, drastically reduced time of response and the possibility to be reused as well. In this study, an array of these sensors has been used inside a portable device called Small Sensor System (S3), described in detail in Section 2.3.

The aim of this work was to find and identify the VOCs set that characterizes *C. jejuni* through Gas-Chromatography Mass-Spectrometry (GC-MS) and to assess the capability of S3 to distinguish between samples inoculated with this microorganism and control specimens to follow *C. jejuni* temporal development. The success of this study can lay the foundation to deepen the research in this field in order to use S3 as a tool for prevention of illness and food quality control in the future. 

## 2. Materials and Methods

### 2.1. Samples Preparation

Samples were prepared using *C. jejuni* subsp. *jejuni* type strain purchased from DMSZ, DSM number 4688, (ATCC 33560, CCUG 11284, CIP 702, NCTC 11351) and Brain Heart Infusion Broth (BHI) media purchased from Sigma Aldrich (Merck). Tubes containing 9 mL of sterile BHI were inoculated with *C. jejuni* cells and were aerobically incubated for 24 h at 35 °C in order to produce enough biomass to proceed with the next step of analysis. After the incubation, the culture was used to inoculate tubes of sterile BHI media in order to realize the same optical density (OD) of the number 3 standard of McFarland that corresponds to a concentration of 9 × 10^8^ CFU (Colony Forming Unit) by mL [24]. Subsequently, serial dilutions using sterile BHI media were performed until the concentration was decreased by 4 orders of magnitude to 9 × 10^4^ CFU/mL. This concentration was used for the inoculation of the analyzed vials.

Sterile chromatography 20 mL vials containing 4 mL of BHI were inoculated with 100 μL of the 9 × 10^4^ CFU/mL solution reaching a final concentration of 2.20 × 10^3^ CFU/mL. Control vials were performed as well keeping the vials containing 4 mL of BHI with no inoculum. Furthermore, in order to control the effective number of cells at the beginning and the end of the analysis, a plate count technique was applied using four plates for each time (T0 and T20).

### 2.2. GC-MS Analysis

The Gas Chromatograph (GC) used during the analyses was a Shimadzu GC2010 PLUS (Kyoto, KYT, Japan), equipped with a Shimadzu single quadrupole Mass Spectrometer (MS) MS-QP2010 Ultra (Kyoto, KYT, Japan) and an autosampler HT280T (HTA s.r.l., Brescia, Italy). The GC-MS analysis was coupled with the Solid-Phase Micro Extraction (SPME) method in order to find the most characteristic VOCs for each sample.

The fiber used for the adsorption of volatiles was a DVB/CAR/PDMS-50/30 µm (Supelco Co., Bellefonte, PA, USA). The fiber was exposed to the headspace of the vials after heating and shaking the samples in the HT280T oven, thermostatically regulated at 40 °C for 15 min, with the aim of creating the headspace equilibrium. The length of the fiber in the headspace was kept constant. Desorption of volatiles took place in the injector of the GC-MS for 6 min at 240 °C.

The gas chromatograph was operated in the direct mode throughout the run, with the mass spectrometer in electron ionization (EI) mode (70 eV). GC separation was performed on a MEGA-WAX capillary column (30 m × 0.25 mm × 0.25 μm, Agilent Technologies, Santa Clara, CA, USA). Ultrapure helium (99.99%) was used as the carrier gas, at the constant flow rate of 1.5 mL/min. The following GC oven temperature programming was applied. At the beginning, the column was held at 40 °C for 3.5 min, and then raised from 40 to 90 °C at 5 °C/min. Next, the temperature was raised from 90 °C to 220 °C, with a rate of 12 °C/min; finally, 220 °C was maintained for 7 min.

The GC-MS interface was kept at 200 °C. The mass spectra were collected over the range of 40 to 500 *m*/z in the Total Ion Current (TIC) mode, with scan intervals at 0.3 s. The identification of the volatile compounds was carried out using the NIST11 and the FFNSC2 libraries of mass spectra.

Four samples were analyzed: control and *C. jejuni* at times T0 and T20.

### 2.3. S3 Analysis

The S3 device used in the present work has been completely designed and constructed at SENSOR Laboratory (University of Brescia, Brescia, Italy) in collaboration with NASYS S.r.l., a spin-off of the University of Brescia, Brescia, Italy. It has been described in other works [25,26,27,28]. Briefly, the tool comprises three parts: Pneumatic components that transfer VOCs from the headspace of samples to the sensing chamber; electronic boards that manage the acquisition and transmission of the data from the device to the dedicated Web-App and allow the synchronization between S3 and the auto-sampler; and a sensing chamber, that can host from five to ten different metal oxide (MOX) gas sensors and is thermostatically isolated in order to avoid any influence of the surrounding environment. To function properly, sensors need a reference value that has been obtained by filtering the ambient air with a small metal cylinder (21.5 cm in length, 5 cm in diameter) filled with activated carbons.

Eight MOX gas sensors were used. Three of them are MOX nanowire [29,30]. Two of them are tin oxide nanowires sensors, both grown by means of the Vapor Liquid Solid technique [31], using a gold catalyst on the alumina substrate and functionalizing one of them with gold clusters; the third sensor has an active layer of copper oxide nanowires. The working temperature is 350 °C, 350 °C and 400 °C, respectively. The other three sensors are prepared with Rheotaxial Growth and Thermal Oxidation (RGTO) thin film technology [32]; one is tin oxide functionalized with gold clusters (working at 400 °C), while the other two are pure tin oxide (working at 300 °C and at 400 °C, respectively). The last two are commercial MOX sensors produced by Figaro Engineering Inc. (Osaka, Japan). In particular, they are the TGS2611 and TGS2602, which are sensitive to natural gases and odorous gases like ammonia, respectively, according to the datasheet of the company. Commercial sensors have been mounted on our e-nose in order to evaluate the performances of nanowire sensors. Details of S3 sensors made at SENSOR Laboratory are summarized in Table 1. Response to 5 ppm of ethanol, selectivity (response ethanol/response carbon monoxide) and limit of detection (LOD) of ethanol are also included.

The autosampler headspace system HT2010H (HTA s.r.l., Brescia, Italy) was coupled with S3. It supports a 42-loading-sites carousel and a shaking oven to equilibrate the sample headspace. 40 vials were placed in a randomized mode into the carousel. Among these vials, 5 were control samples analyzed at times 2.5 h, 5.5 h, 8.5 h, 11.5 h and 14.5 h. Each vial was incubated at 40 °C for 10 min in the autosampler oven. The sample headspace was then extracted from the vial in the dynamic headspace path and released into the carried flow (100 sccm). The analysis timeline can be divided into three different steps for a duration of 30 min per sample divided as follows: 5 min to analyze samples, 5 min to clean the tube that carries VOCs from sample headspace to sensing chamber and 20 min to restore the baseline. Thanks to the processor integrated in the S3 instrument, the frequency at which the equipment works is equal to 1 Hz. 

### 2.4. S3 Data Analysis

Data analysis was performed using MATLAB^®^ R2015a software (MathWorks, Natick, MA, USA). First of all, sensors responses in terms of resistance (Ω) were normalized when compared to the first value of the acquisition (R_0_). For all the sensors, the difference between the first value and the minimum value during the analysis time was calculated; hence, ΔR/R_0_ has been extracted as featured.

Principal Component Analysis (PCA) was applied to this data matrix in order to evaluate the ability of the system to follow variation over time of the number of bacteria and therefore also the quantity of VOCs emitted. 

## 3. Results and Discussion

### 3.1. GC-MS Results

Chromatograms of analyzed samples were compared to highlight differences between control samples and *C. jejuni* ones to see the variation of emitted VOCs in the headspace between T0 and T20.

The comparison between control and *C. jejuni* specimens at time T0 underlines no significant differences in terms of number and amount of VOCs. In Table 2, the list of compounds with their retention time (RT) and abundance in arbitrary unit is reported. Correlation coefficient has been calculated to get how similar the two samples were; a value equal to 0.9965 has been obtained. This proves that during the conditioning period of 15 min before fiber exposure in the GC injector, *C. jejuni* VOCs production was too small to change headspace composition.

On the contrary, sample analysis after 20 h has indicated changes in vial headspace due to microorganisms metabolic activity and to slow release of VOCs contained in BHI broth. In Table 3, the list of VOCs is shown. Main differences between the specimens reside in the presence of alcohol compounds, such as 1-pentanol, acetoin, 2,7-dimethyl-4,5-octanediol, 2-propyl-1-pentanol, bicyclo[3.2.1]octan-6-ol, 1-nonanol, γ-methylmercaptopropyl alcohol and (9*E*)-9-hexadecen-1-ol greater in *C. jejuni* samples than control one. This result points out how sugar fermentation went on during a 20 h incubation period at 37 °C. Furthermore, this heating phase could also be responsible for the formation of new compounds derived from pyrazine, like trimethylpyrazine and 2-ethyl-3,6-dimethylpyrazine. In this case, the correlation coefficient is equal to 0.2666, indicating the samples were strongly diverse.

Growth of *C. jejuni* is also confirmed by microbiological analysis. There were (8.57 ± 1.18) × 10^4^ CFU/mL at time T0 in terms of mean ± standard deviation calculated on four plates and (1.38 ± 0.40) × 10^7^ CFU/mL at time T20.

### 3.2. S3 Results

**Figure 1:**
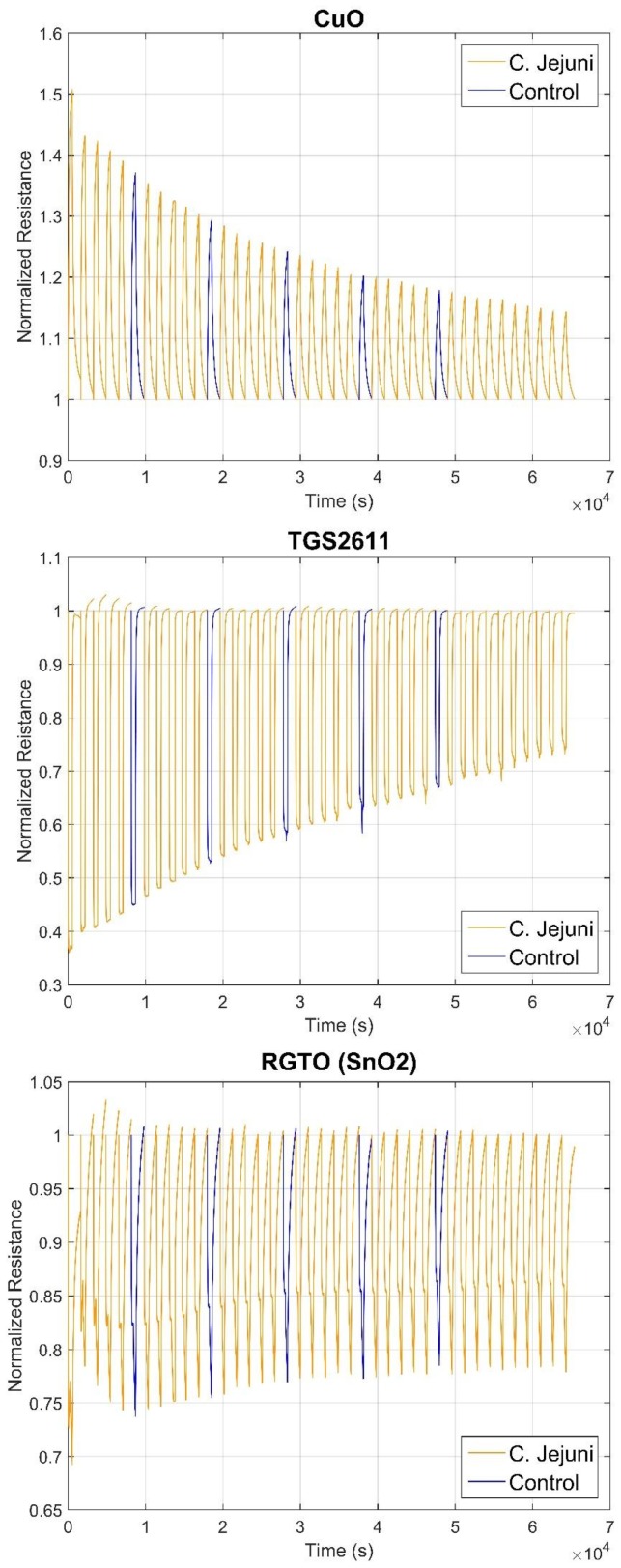
Resistance variations of three sensors once exposed to VOCs. From top to bottom: copper oxide nanowire, TGS2611 and tin oxide Rheotaxial Growth and Thermal Oxidation (RGTO). On the *x*-axis there is time in seconds, on the *y*-axis normalized resistance.

First step of S3 data analysis consisted of checking which of the eight sensors were performing more. Sensor responses were normalized in order to highlight the variation of the resistance once sensing materials were exposed to VOCs. Five sensors showed the best performances: two RGTO (SnO_2_Au and SnO_2_ heated at 300 °C), SnO_2_Au nanowire, copper oxide and TGS2611. Resistance variation over time for all measures is shown in Figure 1 for three kinds of sensors, i.e., RGTO, nanowire and commercial MOX.

CuO sensing material exhibits an increase in resistance with respect to the R_0_ value, while TGS2611 and SnO_2_ RGTO have an opposite behavior due to their n-type semiconductor characteristic. However, all of them are characterized by the decreasing of ΔR with the growth of time. This trend is more evident for CuO and TGS2611 sensors. Conversely, RGTO has an increase of ΔR for the first five samples, while from the seventh specimen it has the same kind of response as the other two, even if it is less accentuated. This tendency could be explained considering that the number of microorganisms grows over time very quickly; it has been shown that they double their number in BHI broth in 75 min (average value) [33]. Hence, many VOCs could be used by *C. jejuni* to feed, thus subtracting them from the headspace. At the same time, new compounds are emitted from bacteria and pass in the gaseous phase, as shown in Table 2 and Table 3. It is important to underline that for 20 h not only the alcohols have increased in number and amount, but also other compounds. Among them, the one present in greater quantity is pyrindan, a bicyclic compound containing a pyridine ring. The reduction of ΔR/R_0_ value could be due to action of this compound. Furthermore, it can be noticed that the first sample of the analysis produced a ΔR significantly different in respect to the others; it is higher for all sensors, especially for RGTO and nanowire sensors. Since this could be the result of a different conditioning, it has been discarded for the following analysis.

**Figure 2:**
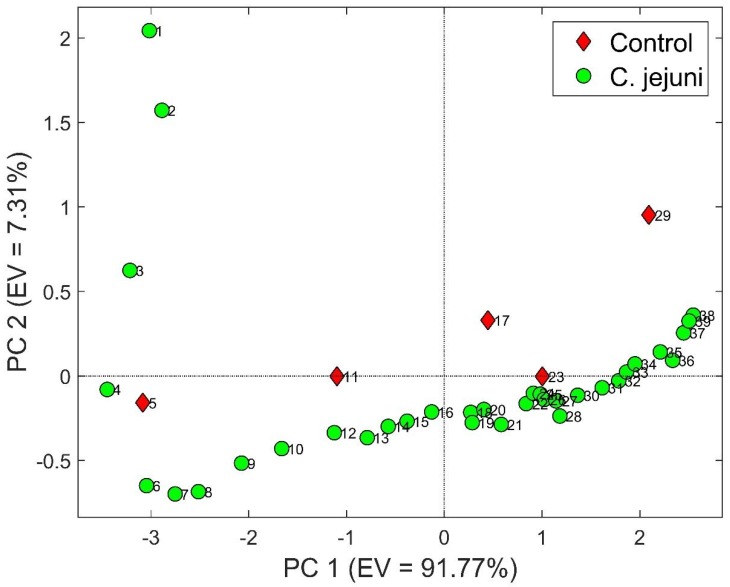
Principal Component Analysis (PCA) done with first two components (total variance equal to 99.08%). Green circles are *C. jejuni* samples, red diamond control ones.

Figure 2 refers to PCA that has been performed using the five aforementioned sensors. The first two Principal Components (PC) were used for a total explained variance equal to 99.08% (91.77% in PC1 and 7.31 in PC2). It is possible to identify two different trends. For *C. jejuni* samples, first four samples (0.5–2.5 h) are characterized by descending scores along PC2 axis, while the other specimens (3–20 h) are distributed essentially along PC1 ascending scores. Furthermore, the distance between points decreases as time goes on and it can be explained by the typical growth curve of microorganism. Indeed, it is characterized by four phases: (A) lag phase (bacteria adapt themselves to growth conditions and are yet not able to divide), (B) log phase (cell doubling), (C) stationary phase (growth rate and stationary rate are equal due to a growth-limiting factor such as the depletion of an essential nutrient) and (D) death phase (bacteria die). In PCA, lag phase corresponds to the first four points that move along PC2 axis, log phase to the following eleven samples (3–8 h) and stationary phase to the remaining ones (8.5–20 h). Instead, control samples assume higher scores along both PCs axes with increasing time. It is due to the slow release in the headspace of some VOCs contained in the BHI broth. The only exception is the fourth control sample that does not follow the parabolic trend of the others, but it is closer to *C. jejuni* points.

## 4. Conclusions

This work demonstrates the potential of this technology for the development of a device able to give a response very quickly when compared to classical microbiological methods, 5 min for the former and several hours for the latter. This lays the foundation to develop a rapid and sensitive detection method for *C. jejuni* in the future. Innovative gas sensors with nanowire and RGTO morphologies used in this study have proven to be useful tools for the identification and characterization of microbiological contamination. In particular, PCA done with five sensors shows the capability of the system to follow bacteria growth along a period of 20 h. During which the sensors used were able to associate the response faithfully following the growth curve of the contaminated microorganisms. We will plan to continue this study by focusing on reducing the detection threshold in order to use this tool to individuate the presence of *C. jejuni* at low concentrations and to avoid human infections. We will evaluate in future works how the food matrix where *C. jejuni* develops and grows will influence sensor responses and their LODs.

## Figures and Tables

**Table 1:** Type, composition, morphology, operating temperature, response (ΔR/R), selectivity (response ethanol/response carbon monoxide) and limit of detection (LOD) of ethanol for S3 sensors made at the SENSOR Laboratory.

Materials (Type)	Composition	Morphology	Operating Temperature (°C)	Response to 5 ppm of Ethanol	Selectivity	Limit of Detection (LOD) of Ethanol (ppm)
SnO_2_Au (n)	SnO_2_ functionalized with Au clusters	RGTO	400 °C	6.5	3	0.5
SnO_2_ (n)	SnO_2_	RGTO	300 °C	3.5	2.5	1
SnO_2_ (n)	SnO_2_	RGTO	400 °C	4	2	0.8
SnO_2_Au + Au (n)	SnO_2_ grown with Au and functionalized with gold clusters	Nanowire	350 °C	7	2.5	0.5
SnO_2_Au (n)	SnO_2_ grown with Au	Nanowire	350 °C	5	2.1	1
CuO (p)	CuO	Nanowire	400 °C	1.5	1.5	1

**Table 2:** List of volatile organic compounds (VOCs) for *C. jejuni* and control samples with their retention times (RT) and abundance in arbitrary units at time T0.

RT	VOC	Abundance	
		*Campylobacter*	Control
1.552	3-Butynol	5,488,041	5,289,186
2.674	Isovaleraldehyde	28,336,125	25,535,856
5.386	Dimethyl Disulfide	5,401,037	6,048,406
8.666	3-*O*-Methyl-d-fructose	912,105	904,062
9.281	1-Hydroperoxyheptane	533,178	418,838
12.332	2,5-Dimethylpyrazine	623,139	560,597
14.432	Nonanal	512,867	227,918
14.624	6-Methyloctadecane	549,617	794,739
15.419	4-Methyl-2-oxovaleric acid	457,664	412,957
15.805	2-Acetylamino-3-hydroxy-propionic acid	27,861	51,993
16.017	1-(2-Methoxy-1-methylethoxy)-2-propanol	519,216	240,149
16.372	Ethylhexanol	404,819	389,367
16.866	Benzaldehyde	12,509,648	13,515,034
17.430	3-Trifluoroacetoxydodecane	92,701	168,299
18.455	3-Hydroxycyclohexanone	145,875	206,891
18.695	Acetophenone	2,201,980	1,987,623
19.545	[(2-Ethylhexyl)methyl]oxirane	93,105	281,168
19.950	Methoxy-phenyl-oxime	1,545,714	1,301,333
20.871	Heptanoic acid	268,343	304,130
21.205	Benzyl alcohol	280,516	246,860
21.555	2-[2-(Benzyloxy)-1-(1-methoxy-1-methylethoxy)ethyl]oxirane	226,967	202,114
22.021	1-Dodecanol	222,887	482,480
22.465	Phenyl carbamate	51,364	93,104
23.580	Octanal	121,544	130,790
24.480	Octadecanal	131,841	76,150
25.078	2,6-Bis(tert-butyl)phenol	691,529	597,086
27.565	N,N-Dimethylformamide ethylene acetal	53,073	24,830

**Table 3:** List of VOCs for *C. jejuni* and control samples with their retention times (RT) and abundance in arbitrary units at time T20.

RT	VOC	Abundance	
		*Campylobacter*	Control
1.540	3-Butynol	8,834,835	2,135,579
2.266	Isovaleraldehyde	221,889,809	3,852,176
4.142	Dimethyl Disulfide	42,167,678	0
8.377	1-Pentanol	236,112,172	0
9.229	Isoamyl Alcohol	0	737,186
10.673	Acetoin	1,974,910	0
11.125	2-Methylbutyl isovalerate	1,684,776	0
12.299	2,5-Dimethylpyrazine	0	693,770
14.400	Nonanal	0	123,487
14.483	Trimethylpyrazine	0	258,896
14.600	6-Methyloctadecane	0	60,501
15.275	2-Ethyl-3,6-dimethylpyrazine	243,733	0
15.392	4-Methyl-2-oxovaleric acid	454,870	273,645
15.702	Ammonium acetate	812,172	572,057
15.903	2,7-Dimethyl-4,5-octanediol	945,696	0
16.186	1-(2-Methoxy-1-methylethoxy)-2-propanol	0	79,930
16.295	2-Propyl-1-pentanol	308,880	0
16.340	Ethylhexanol	267,747	301,354
16.845	Benzaldehyde	0	14,760,479
17.366	1-Octanol	972,272	256,675
17.550	Bicyclo[3.2.1]octan-6-ol	164,638	0
17.946	2-Undecanone	103,647	0
18.419	3-Hydroxycyclohexanone	0	79,109
18.581	Benzeneacetaldehyde	5,087,169	0
18.668	Acetophenone	0	669,190
18.740	1-Nonanol	1,331,499	0
18.914	Methyl 4-hydroxybutanoate	0	204,876
19.446	γ-Methylmercaptopropyl alcohol	690,884	0
19.923	E-11,13-Tetradecadien-1-ol	2,393,731	532,815
20.575	β-Phenethyl acetate	131,503	0
20.844	Heptanoic acid	488,771	215,935
21.187	Benzyl alcohol	342,619	146,035
21.540	Phenylethyl Alcohol	26,577,900	1,429,647
21.750	m-Tolunitrile	0	60,783
21.996	1-Dodecanol	492,208	270,053
22.316	Tropone	165,612	56,068
22.443	4-Hydroxybenzenephosphonic acid	0	77,574
22.684	Nerolidyl acetate	0	116,357
22.876	Octanoic acid	178,530	73,913
23.555	1,3,2-Dioxaborolane, 2-ethyl-4-(3-oxiranylpropyl)-	0	48,973
23.822	(9*E*)-9-Hexadecen-1-ol	188,001	0
25.047	2,4-Di-tert-butylphenol	204,650	200,860
26.632	Pyrindan	103,007,890	14,885,449
27.555	N,N-Dimethylformamide ethylene acetal	40,116	40,546
